# West Texas Medical Association

**Published:** 1902-09

**Authors:** W. B. Russ

**Affiliations:** Secretary


Secretary.

### West Texas Medical Association.

A special meeting of the West Texas Medical Association was held July 31, 1902, at New Braunfels, Texas.

Vice-President Dr. T. T. Jackson called the session to order at 3:30 p. m. Dr. Garwood, on behalf of the local fraternity, welcomed the association and its visitors. In response Dr. Jackson thanked Dr. Garwood and his associates on the local committee for the splendid entertainment they had provided.

Dr. M. L. Graves, of San Antonio, presented two cases of "Peripheral Neuritis" for clinical study. In his remarks Dr. Graves said that the frequent association of psychoses with peripheral neuritis was to him an extremely interesting subject for study.

Dr. J. H. Reuss, of Cuero, suggested that the presence of some previously existing pathological condition would explain the mental disturbances noted in such cases.

Drs. King and Lankford, of San Antonio, also took part in the discussion. The latter inquiring of Dr. Graves if many such cases are not traumatic in origin.

Dr. H. N. Graves, of Georgetown, read an interesting and instructive paper on "Serum Therapy," in which he referred especially to the great reduction in mortality from diphtheria since the introduction of antitoxin. Dr. Lankford led the discussion. He agreed with Dr. Graves that the development of serum therapy deserves to rank as one of the greatest achievements of modern medicine.

Drs. F. Paschal, J. H. Reuss and S. Burg also discussed the paper approvingly. All three described cases in which wonderful results had been obtained by the prompt use of diphtheria antitoxin.

Dr. C. E. R. King, of San Antonio, in a spirited and convincing speech took Dr. Graves and the rest severely to task. He expressed the belief that the good results, if any, obtained from the use of a diphtheria antitoxin must be due to the carbolic acid used as a preservative. He characterized the doctrine of serum therapy as absurd and those who believe in it as dupes of the concerns that manufacture the socalled antitoxins.

Dr. Russ called attention to the fact that the statistics used to prove that the introduction of antitoxin has brought about a great decrease in the mortality from diphtheria are misleading. It is impossible to determine a rise or fall in the death rate in this or any similar disease by a comparative study of mortality records during given periods, because of the great variation in the virulence of different epidemics, and because of the faulty methods in which records have been kept. Again by the general use of the microscope many cases of mild diphtheria are now diagnosed and reported which were formerly overlooked.

Dr. F. E. Daniel, of Austin, an honorary member of the society, and chairman of the State Medical Association's Committee on State Board of Health, made an interesting talk on "The Proposed State Board of Health," in which he stated that he had been laboring for twenty years to bring about the establishment of such a board, and that the prospects of success were better now than ever before. Following Dr. Daniel, remarks were made along the same line by State Health Officer George R. Tabor, Dr. A. Nolte, of the Louisiana State Board of Health, and by City Health Officer Frank Paschal, of San Antonio.

At 6:30 the afternoon session adjourned and the entire party of forty-five went for a ride on the Comal river on board the steamer "Wilhelm der Grosse," which had been chartered for the occasion. After the return the party visited the various points of interest in Landa's Park and in the town.

At 8:30 dinner was served at the Platz hotel, during which Dr. G. Graham Watts, of San Antonio, served as toast master, and toasts were responded to by President W. J. Mathews, of the Austin District Medical Society, and Drs. Daniel, Tabor, Nolte, King, Paschal, Reuss, Watts, Graves, Garwood, and others.

At ten o'clock a short evening session was held, at which nine new applications for membership were read by the Secretary, and at which Dr. J. H. Burleson, of San Antonio, read an interesting report of a case of primary mastoiditis. This paper was discussed by Drs. E. F. Hughes and A. J. Nabors, both of San Antonio.

Dr. Watts made some remarks on "Rabies," based on a series of experiments he has recently been conducting in San Antonio and vicinity. These remarks developed a considerable amout of discussion, in which Drs. F. E. Young and Frank Paschal, of San

Antonio, H. N. Graves, of Georgetown, and H. Leonards, of New Braunfels, participated.

The following physicians were present: Drs. F. E. Daniel, W. J. Mathews and W. A. Harper, of Austin; A. Garwood, Noster and H. Leonards, of New Braunfels; T. P. Harrell, of Hunter; C. R. Myrick, of Boerne; L. Hirschfield, of Marion; J. H. Reuss, of Cuero; J. R. Evans, of Devine; D. M. Thurston, of Beeville; W. T. Reeves, of Fisher Store; H. N. Graves, of Georgetown; L. V. Weathers, of Bracken; J. C. Anderson, of Granger; M. B. Grace, of Seguin; M. L. Graves, A. A. Brown, T. P. Lloyd, B. F. Kingsley, F. Paschal, T. T. Jackson, J. S. Lankford, E. T. Hughes, G. Graham Watts, Jas. Bartlett, W. M. Wolff, J. H. Burleson, C. E. R. King, J. P. Oldham, W. B. Russ, Jack Watts, A. J. Nabors, F. E. Young and S. Burg, of San Antonio; Ed. Beall, of San Marcos; A. Nolte, of New Orleans, and State Health Officer G. R. Tabor, of Austin, and R. L. Graham, Shertz.

W. B. Russ, Secretary.

